# Psychological stress and psychological support of Chinese nurses during severe public health events

**DOI:** 10.1186/s12888-022-04451-8

**Published:** 2022-12-19

**Authors:** Yufang Zhou, Youtian Wang, Meilian Huang, Chen Wang, Yuanxin Pan, Junrong Ye, Shengwei Wu, Chenxin Wu, Haoyun Wang, Ting Wang, Aixiang Xiao, Lin Yu

**Affiliations:** 1 The Affiliated Brain Hospital of Guangzhou Medical University (Guangzhou Huiai Hospital), 36 Mingxin Rd, Liwan District, Guangzhou, 510370 China; 2 The Third People’s Hospital of North Guangdong, Shaoguan, China; 3 The Affiliated TCM Hospital of Guangzhou Medical University, 16 Zhuji Rd, Liwan District, Guangzhou, 510310 China

**Keywords:** Nurses, Public health events, Psychological support, Stress

## Abstract

The domestic health care system has been facing a difficult task, especially in medical care, and Chinese nurses are under tremendous psychological pressure. Psychological support is a protective factor to relieve stress. This study examined the stress level and characteristics of Chinese nurses with different psychological support-seeking behaviours. Data from online questionnaires for this cross-sectional study were collected between January 2020 and February 2020 and yielded 2248 valid questionnaires for analysis with a response rate of 99.8%. General information of the respondents was also collected. The nurses’ stress levels were assessed using the Perceived Stress Scale (PSS-10). T tests, chi-square tests, and linear regression were used to examine the relationships among the factors. The results of this survey showed that between January and February 2020, 26.9% of nurses received psychological counselling, and the proportion was higher among men and nurses with lower education. The PSS-10 was related to gender, age group, provincial severity, and confidence in the control of the epidemic. The results showed that psychological support can effectively improve the confidence of domestic nurses in the face of arduous work and effectively relieve the psychological pressure caused by a heavy workload.

**Supplementary Information:**

The online version contains supplementary material available at 10.1186/s12888-022-04451-8

## Introduction

In 2020, China and the world were facing a serious public health event, during which medical workers were engaged in the dual tasks of disease prevention and treatment [1]. Serious public health events lead to heavy workloads and mental burdens among nurses, and these negative impacts influence their work performance [2]. However, very few studies have unveiled the effects of the COVID pandemic with its repeated and ongoing stressors and traumatization among nurses.

Stress refers to a psychological state of tension that happens when an individual’s adaptive capacity does not meet the perceived environmental demands. Robbins’s stress model divides stressors into environmental, organizational, and personal factors that interact with individual differences to produce stressful experiences [3]. Compared with other occupations, nursing is considered to be a profession with a moderate stress level [2, 4]. Clinical registered nurses were the subjects of this study and were facing a greater than normal workload during a public health event outbreak. An increased workload and dangerous working environment can lead to negative emotions, increased psychological stress, and impaired physical health among nurses. These problems can prevent nurses from providing high-quality care, and their work efficiency can be greatly reduced. A study in China found that mental stress among nurses has increased while mental health has declined over the past 19 years, with work and family stress as the main stressors [5]. Jin (2015) showed that 76% of the nurses surveyed felt very stressed at work. Stress can reduce nurses’ work efficiency and reduce their quality of life [6].

The sources of stress vary according to the situation [3]. Previous studies have suggested that fear, separation anxiety, and fear of death were sources of stress for medical workers during the SARS epidemic [7]. Social isolation and high-intensity work were the main sources of stress for front-line medical workers during the Middle East respiratory syndrome (MERS) epidemic [8]. Furthermore, studies have shown that medical workers who participated in the provision of first-line treatment during the epidemic had a higher rate of posttraumatic stress disorder (PTSD) than those who did not participate in the provision of first-line treatment [9].

Psychological guidance and counselling are both means of psychological support, which plays a regulatory role in emotions and has a significant impact on brain activity [10]. Psychological support methods can be classified according to the theoretical model, the treatment object, the treatment form and the treatment time. According to the theoretical model, psychological support can be classified as analytical psychological therapy, cognitive psychotherapy, supportive psychotherapy, behavioural psychotherapy, and interpersonal psychotherapy [11]. Jiao showed that psychological support could mitigate the negative effects of stress and improve work efficiency [12]. During the outbreak of domestic public health events in 2020, the sources of psychological stress for nurses were not clear, and it is not clear whether psychological support can effectively alleviate the psychological pressure on nurses. Generally, psychological stress comes from work, family and society. Therefore, we assume that during a public health outbreak, the psychological stress on nurses stems from family, work and society, as well as anxiety about the public health events, and we assume that psychological support can relieve stress [3, 13]. The objective of this study was to investigate the effect of psychological support on nurse stress levels during a public health outbreak.

## Methods

### Study design

This was a cross-sectional study using a convenience sampling method to investigate registered nurses from a professional nursing group in China. Prior to this study, ethics approval was obtained from the IRB of the Affiliated Brain Hospital of Guangzhou Medical University (approval number: 2020–009). In this study, an online platform was used to collect data (https://www.wjx.cn). The background, purpose, inclusion criteria, and informed consent were displayed before administering the questionnaire. If respondents agreed to participate and clicked the button to indicate their consent, the platform activated the questionnaire automatically. A brief introduction of informed consent was displayed first, and respondents who agreed to participate would click the “*I agree*” button to move to the formal questionnaire. In addition, we did not send these questionnaires to those under 18 years old. In the screening process, if the questionnaire was answered by minors under 18 years old, we eliminated it. We confirmed that all methods of this study were carried out in accordance with relevant guidelines and regulations in the Declaration of Helsinki - ‘Ethics approval and consent to participate’ section.

### Participants

During participant selection, the inclusion criteria were as follows: a) being a nursing care provider, b) being aged 18 to 55 years old, and c) having worked in China in the past 6 months. This study was conducted from January 2020 to February 2020. Survey data were collected as follows. First, the questionnaire was uploaded to an online survey platform with a permanent link. Second, the link was sent to the work chat groups of the Psychiatric Nursing Association (PNA) and the Mental Health Care Commission (MHCC). This participant pool was chosen because both the PNA and MHCC have rigorous criteria for membership; usually, PNA/MHCC members are hospital directors and nursing managers. Third, PNA/MHCC members were invited to participate and share the survey with their colleagues. To optimize the response rate, the link was distributed once per week during the study’s timeframe. The study results were not disseminated to participants.

### Study quality control

To eliminate duplicate submissions, participants were required to link their personal WeChat account (a social application) with the online survey platform before completing the questionnaire. Each WeChat account was only allowed to submit one completed survey. To avoid incomplete data, the questionnaire could only be submitted once all of the items had been answered. Nevertheless, the researchers screened each questionnaire. After careful discussion among the three researchers, four implausible questionnaires were eliminated. The exclusion criteria were a) participants under age 18 and b) the number of working years exceeded the participant’s age.

### Patient and public involvement

In total, we received 2252 questionnaires. After screening and excluding four invalid questionnaires (three where the participant < 18 years and one with an incorrect record of working years), there were 2248 valid questionnaires comprising the final dataset, with an effective questionnaire ratio of 99.8%.

### Measurements

#### General information

Information about the participants’ characteristics was collected, including gender, age, marital status, education level, professional title, working years, province of employment (to determine the severity of the public health incident outbreaks), residence type, confidence in prevention and control of the epidemic and level of psychological support.

#### Stress test scale

**Fig. 1:**
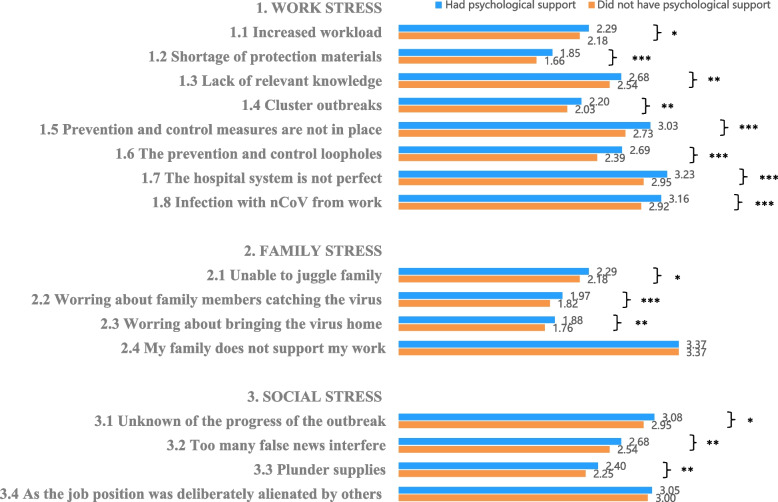
The bar value represent the average of the choices in the item. The high the score,the more participant disagree with the satement. That is, the higher the score, the less stress the participant experienced due to the item. * *P*<0.5，** *P*<0.01，*** *P*<0.001

A self-designed scale (from Item 1.1 to Item 3.4; see Fig. 1) was used to evaluate the source of stress and the PSS-10 scale was used to determine the level of stress.

Based on the Robbins stress model [3], we designed a psychological assessment scale to examine psychological stress during a public health event outbreak, but the scale still needs to be externally validated. Our self-designed scale evaluates the sources responsible for causing stress, including work, family, and social stress factors (from Item 1.1 to Item 3.4; see Fig. 1). The scale contains 16 items, and each item has five options (1 = strongly agree, 2 = agree, 3 = neutral, 4 = disagree, and 5 = strongly disagree). The higher the score, the lower the stress level. The total Cronbach’s alpha of this scale was 0.907, and the Cronbach’s alpha of the work section, family section, and social section was 0.871, 0.717, and 0.822, respectively. Confirmatory factor analysis results showed that a) the AVE values of the work section, family section, and social section were 0.491, 0.625, and 0.545, respectively; b) the CR values of the work section, family section, and social section were 0.878, 0.833, and 0.827, respectively.

The Chinese version of the PSS-10, introduced by Wang et al. (2015), was employed. Participants were asked to respond on a 5-point Likert scale (1 = never, 2 = rarely, 3 = sometimes, 4 = often, and 5 = always) to a total of ten items reflecting the frequency of their stress symptoms [14, 15]. The higher the score, the greater the stress level. On this scale, Items 4, 5, 7, and 8 are positive factor items, and the rest are negative factor items. The results for the positive factor items were scored in reverse. Finally, we reversed the scale to ensure the same question order as in the previous questionnaire. On this premise, a reliability test for the PSS-10 questionnaire was carried out, with a total Cronbach’s alpha of 0.622, and the Cronbach’s alphas of the positive factors and negative factors were 0.840 and 0.821, respectively. The total score for the individual item correlation coefficients ranged between 0.406 and 0.648. Confirmatory factor analysis showed that a) the AVE values of the positive factors and negative factors were 0.584 and 0.664, and b) the CR values of the positive factors and negative factors were 0.846 and 0.922, respectively.

The model fitting of the confirmatory factor analysis for the overall model was as follows: *χ2/df* = 2.870, RMSEA = 0.029, GFI = 0.982, AGFI = 0.966, CFI = 0.990, IFI = 0.990, TLI = 0.981.

### Statistical methods

All statistical analyses were performed using SPSS version 22.0. A *P* value of 0.05 was considered statistically significant. The rate of acceptance of psychological support was expressed as a percentage. The continuous or categorical variables were tested using an independent sample t- or chi-square test. Subsequently, univariate analysis of covariance was used to further observe the effect of psychological support. Among them, psychological support was set as the fixed factor, and gender, age, province of employment, marital status, education level, professional title, working years, dwelling state, and confidence in prevention and control of the epidemic were set as covariates.

## Results

### The frequency of accessing psychological support and the relationship between psychological support and stress

Table 1 indicates that the frequency of accessing psychological support among the nurses was 26.9% (605/2248). The frequency of accessing psychological support was higher among male nurses and nurses with a lower education level (32.4% vs. 25.8%, *χ2* = 7.030, *P* < 0.01; 29.8% vs. 24.6%, *χ2* = 7.537, *P* < 0.01, respectively). Table 2 shows that the work, family, and social stress factor scores and the total score for the self-designed scale for nurses who accessed psychological support were higher than those of nurses who did not access psychological support (*t* = − 5.753, *P* < 0.001; *t* = − 2.587, *P* < 0.001; *t* = − 2.595, *P* < 0.05; *t* = − 4.745, *P* < 0.001, respectively). Furthermore, the positive factor score of nurses who accessed psychological support was higher than that of those who did not access psychological support (*t* = − 2.950, *P* < 0.01). Nurses who received psychological support were more likely to show confidence and emotional self-management (see Fig. 1). In addition, psychological support had a different influence on different stressors (see Fig. 1).

**Table 1:** The relationship between psychological support and demographic data

Demographic data	Psychological support		***χ***^***2***^	***P***
	Yes (***n*** = 605)	No (***n*** = 1643)		
**Gender**				
Woman	483 (25.8)	1389 (74.2)	7.030	0.008
Male	122 (32.4)	254 (67.6)		
**Age group**				
18 ~ 29 years	290 (28.2)	737 (71.8)	4.065	0.131
30 ~ 39 years	199 (24.4)	616 (75.6)		
40 ~ 49 years	116 (28.6)	290 (71.4)		
**Marital status**				
Unmarried	219 (27.9)	566 (72.1)	1.083	0.582
Married	386 (26.5)	1077 (73.5)		
Other	6 (20.7)	23 (79.3)		
**Education level**				
Under bachelor	297 (29.8)	700 (70.2)	7.537	0.006
Bachelor or above	308 (24.6)	943 (75.4)		
**Professional title**				
Unlicensed nursing assistant	78 (26.4)	218 (73.6)	1.016	0.602
Primary	336 (27.8)	874 (72.2)		
Intermediate or above	191 (25.7)	551 (74.3)		
**Working years group**				
0 ~ 2 years	95 (29.7)	225 (70.3)	3.364	0.499
3 ~ 5 years	129 (28.4)	326 (71.6)		
6 ~ 9 years	130 (26.7)	356 (73.3)		
10 ~ 19 years	151 (24.6)	462 (75.4)		
Above 20 years	100 (26.7)	274 (73.3)		
**Province**				
Hubei province	13 (19.1)	55 (80.9)	2.166	0.141
Not Hubei province	592 (27.2)	1588 (72.8)		
**Dwelling state**				
Live alone	124 (30.8)	279 (69.2)	3.713	0.054
Not live alone	481 (26.1)	1364 (73.9)		
**Confidence in prevention and control of the epidemic**				
Have confidence	512 (29.5)	1222 (70.5)	26.351	<.001
Lack of confidence	93 (18.1)	421 (81.9)

**Table 2:** The relationship between psychological support and stress

Psychological stress score	Psychological support		***t***	***P***
	Yes ($\overline{X}$ ± *SD*)	No ($\overline{X}$ ± *SD*)		
**Self-designed Scale score**				
Work stress score	21.13 ± 6.531	19.40 ± 5.686	−5.753	<.001
Family stress score	9.50 ± 3.127	9.13 ± 2.684	−2.587	<.001
Social stress score	11.21 ± 3.857	10.75 ± 3.461	−2.595	0.010
Total score	41.85 ± 11.763	39.29 ± 10.152	−4.745	<.001
**PSS-10 score**				
Positive factor score	12.18 ± 3.638	11.68 ± 3.296	−2.950	0.003
Negative factor score	14.53 ± 3.634	14.30 ± 3.572	−1.318	0.188
Total score	26.75 ± 4.472	26.61 ± 4.685	0.647	0.518

These results show that during an outbreak of public health events, the majority of nurses seek psychological support, which can reduce stress and regulate their confidence.

### Factors influencing stress during the public health event outbreak

In general, education level (*F* = 12.922, *P* < 0.001), confidence in control of the epidemic (*F* = 76.948, *P* < 0.001), and psychological support (*F* = 16.551, *P* < 0.001) were the overall factors influencing stress during public health event outbreaks. For work stress specifically, the influential factors were related to gender (*F* = 4.424, *P* < 0.05), education level (*F* = 10.081, *P* < 0.01), confidence in controlling the epidemic (*F* = 86.005, *P* < 0.001), and psychological support (*F* = 26.431*P* < 0.001). For family stress, the influential factors were related to age (*F* = 5.650, *P* < 0.05), marital status (*F* = 17.611, *P* < 0.001), and confidence in controlling the epidemic (*F* = 41.725, *P* < 0.001). For social stress, the influential factors were related to confidence in controlling the epidemic (*F* = 31.004, *P* < 0.001) and psychological support (*F* = 5.311, *P* < 0.05) (see Table 3). Gender (*F* = 4.287, *P* < 0.05), age (*F* = 5.521, *P* < 0.05), province (*F* = 11.827, *P* < 0.01), and confidence in controlling the epidemic (*F* = 80.701, *P* < 0.001) were the factors influencing stress perception. Positive stress feelings were influenced by gender (*F* = 6.194, *P* < 0.05), age (*F* = 6.627, *P* < 0.05), professional title (*F* = 26.261, *P* < 0.001), and psychological support (*F* = 8.089, *P* < 0.01), while negative stress feelings were influenced by gender (*F* = 14.774, *P* < 0.001), province (*F* = 8.586, *P* < 0.01), marital status (*F* = 5.017, *P* < 0.05), professional title (*F* = 8.997, *P* < 0.05), and confidence in controlling the epidemic (*F* = 85.257, *P* < 0.001) (see Table 4).

**Table 3:** Univariate covariance analysis of factors related to elevated self-designed scale

Variables	Self-designed Scale score			Work stress score			Family stress score			Social stress score		
	***F***	Observed Power	***P***	***F***	Observed Power	***P***	***F***	Observed Power	***P***	***F***	Observed Power	***P***
Psychological support	16.551	0.982	<.001	26.431	0.999	<.001	3.619	0.477	0.057	5.311	0.634	0.021
Gender	3.420	0.456	0.065	4.424	0.557	0.036	0.568	0.117	0.451	2.094	0.304	0.148
Age group	0.003	0.050	0.958	1.492	0.231	0.222	5.650	0.661	0.018	1.240	0.200	0.266
Marital status	–	–	–	0.474	0.106	0.491	17.611	0.987	<.001	0.000	0.050	0.998
Education level	12.922	0.949	<.001	10.081	0.888	0.002	3.448	0.459	0.063	–	–	–
Professional title	–	–	–	0.669	0.129	0.414	1.474	0.228	0.225	–	–	–
Working years group	0.065	0.057	0.799	1.027	0.173	0.311	0.279	0.082	0.597	1.116	0.184	0.291
Province	1.425	0.222	0.233	1.259	0.202	0.262	0.315	0.087	0.575	1.261	0.202	0.262
Dwelling state	0.264	0.081	0.607	0.766	0.141	0.382	0.234	0.077	0.629	0.930	0.161	0.335
Confidence in prevention and control of the epidemic	76.948	1.000	<.001	86.005	1.000	<.001	41.725	1.000	<.001	31.004	1.000	<.001

**Table 4:** Univariate covariance analysis of factors related to elevated PSS-10

Variables	PSS-10 score			Positive factor score			Negative factor score		
	***F***	Observed Power	***P***	***F***	Observed Power	***P***	***F***	Observed Power	***P***
Psychological support	3.084	0.419	0.079	8.089	0.811	0.004	0.109	0.970	0.741
Gender	4.287	0.544	0.039	6.194	0.701	0.013	14.774	0.073	<.001
Age group	5.521	0.651	0.019	6.627	0.730	0.010	0.202	0.610	0.653
Marital status	2.771	0.384	0.096	0.685	0.131	0.408	5.017	0.107	0.025
Education level	0.311	0.086	0.577	2.956	0.405	0.086	0.484	0.850	0.487
Professional title	0.525	0.112	0.469	26.261	0.999	<.001	8.997	0.298	0.003
Working years group	0.026	0.053	0.871	3.070	0.418	0.080	2.045	0.834	0.153
Province	11.827	0.930	0.001	0.400	0.097	0.527	8.586	0.345	0.003
Dwelling state	0.777	0.143	0.378	0.917	0.160	0.338	2.441	1.000	0.118
Confidence in prevention and control of the epidemic	80.701	1.000	<.001	0.278	0.082	0.598	85.257	1.000	<.001

These results show that gender, age, province, education level, confidence in controlling the epidemic, and psychological support were the main factors influencing stress during the public health event outbreak.

## Discussion

The main findings of this study are as follows. a) During the public health event outbreak, nurses tended to seek psychological support. We found that 26.9% (605/2248) of nurses had sought psychological support during the COVID pandemic, highlighting that this public event had caused critical mental stress among nurses. b) Nurses who accessed psychological support had a greater degree of confidence in the authorities’ ability to control the epidemic than nurses who did not access psychological support. When an individual faces excessive psychological stress, their mental health can affect their work efficiency and work performance. Actively seeking psychological support can improve an individual’s psychological state and adaptability and thus enhance their work performance. c) Nurses with psychological support were more positive in the face of stress, suggesting that their stress perception was positively regulated.

### Factors influencing psychological support

Nurses’ stress perception was affected by the severity of the public health event outbreak in the province where the nurses were located [16, 17]. Fergusson et al.’s (2014) prospective study comparing people in earthquake areas with those in nonearthquake areas found that the proportion of depression and PTSD cases in earthquake areas was significantly higher than that in nonearthquake areas [18]. The severity of psychological stress among nurses is affected by outbreaks of public health events [19–21].

Our results showed that nurses who accessed psychological support had lower levels of perceived stress and more confidence in controlling the epidemic than those who did not access psychological support. However, more studies are needed to explain the causes of help-seeking behaviour and perceived levels of stress, as well as the effectiveness of psychological support in stress relief.

Stress is especially important to an individual’s confidence. Negative emotions experienced during stressful situations may interact with cortisol, affecting confidence [9]. Nurses’ confidence in their clinical skills was found to decrease under stress; however, psychological support can regulate the negative effects of stress, thus enhancing confidence [22]. It is notable that psychological support can regulate people’s negative emotions because under stress, emotional self-management is very important to improve mental resilience [23]. Psychological resilience is considered to be a phenomenon in which a person can adapt and accept challenges in the face of adversity and maintain their mental health [24]. Our results have shown that nurses who accessed psychological support had better control over their emotions. This finding suggests that psychological support can enhance nurses’ psychological resilience. It can help nurses under stress adjust better [9, 22–25].

### Differences between nurses who accessed psychological support and those who did not

Nurses with a lower education level were more likely to seek psychological support than those with a higher education level. Nurses with a lower education level have a lower nursing ability and comprehensive quality, and generally, people with a lower education level experience greater psychological stress. Due to the dual influence of educational stress and external epidemic-related stress, nurses with a lower education level may experience greater stress; therefore, they might be more inclined to seek psychological support [26–28].

Male nurses were also found to be more likely to seek psychological support than female nurses. Many studies have focused on gender differences in the experience of stress. Zhang Yuru and her colleagues conducted a survey of 272 nurses who were working in the emergency departments of five hospitals from January 2018 to June 2019. Among the 272 nurses, there were 76 male nurses (29.01%) and 186 female nurses (70.99%). Influenced by traditional concepts, male nurses often needed to take on more tasks at work. Because their strength and energy were perceived as being higher than that of women, the male nurses were asked to work longer hours and were assigned more difficult tasks. Therefore, male nurses are more likely to experience psychological pressure, and it is especially important for them to seek psychological support [29]. In general, women are more stressed than men [30]. When experiencing stress, women are more likely to use self-help, avoidance, or self-punishment, while men cope rationally or in a detached way [31, 32]. The gender difference regarding dealing with stress may be related to negative interpersonal relationships, demanding jobs, a high degree of competition, gender discrimination, and biological differences [33–38].

The biological mechanisms of stress have been widely studied. In men, stress is associated with the right prefrontal cortex and the left orbitofrontal cortex, while in women, stress activates the limbic system. Studies have also indicated that there are sex differences in the core components of the HPA axis stress response. Serum corticosterone concentration and brain-derived neurotrophic factor methylation also show gender differences. Under controlled stress, it was found that 5-hydroxytryptamine (5-HT) in men’ dorsal raphe nucleus (DRN) was effectively inhibited, while 5-HT in women’s DRN was not involved in behavioural control [39–42]. Thus, men tend to seek psychological intervention when they are aware that stress has influenced their mental status and work performance. In addition, controllable stress can protect against uncontrollable stress in the future, which explains why nurses who accessed psychological support experienced less stress and had a more positive coping attitude [37, 39–45].

### Other common factors that influence stress

Our study found that age, professional title, and marital status affected the nurses’ stress level and stress perception; this finding is similar to previous research results.

The results of the mental health survey that Fan et al. (2019) administered to clinical nurses aged 20 to 49 showed that nurses under 30 years old experienced the greatest psychological stress, followed by nurses over 40 years old [46]. In contrast, Cohen et al. (2012) found that younger and older people were less stressed, while middle-aged people were more stressed [47].

In social and family life, the status, quality, and interaction of marital and professional titles symbolizing social status are closely related to stress. In general, nurses who hold supervisory positions experience the highest levels of work stress and the lowest levels of work satisfaction. Married nurses experienced lower levels of stress and physical discomfort and better social adjustment than unmarried nurses. Our results are in line with previous studies’ findings [38, 48–50].

## Strengths and limitations

This study focused on the psychological stress and psychological support of nurses dealing with public events during the peak period of public health events in China. This study used a self-designed scale to measure the source of stress, which includes three factors: work, family, and society. In the future, we will expand the use of this questionnaire. This study had the following limitations: a) a convenience sampling method was employed, which might affect the generalizability of the conclusion. In the future, more rigorous sampling methods should be adopted to control the sampling deviation; b) other sources of stress might not be included, and more potentially influential stressors should be included in the future; and c) although this questionnaire has passed the reliability and validity tests, the use of this questionnaire is low at present; thus, further examination of the external validity of this questionnaire is needed.

## Conclusion

During a public health event outbreak, nurses experienced high work, family, and social stress, among which gender, age, education level, the severity of public health incidents in the region, confidence in the authorities’ ability to control the epidemic, and psychological support were shown to be different. Psychological support has a benign regulatory effect on nurses, with the potential to improve their confidence and reduce stress. Therefore, it can be concluded that psychological support interventions for nurses during the epidemic is necessary and beneficial. Our findings suggest that society should pay attention to the mental health of nursing staff in addition to their physical health during public health events.

## Supplementary Information

**Additional file 1.** Medical staff respond to the investigation of the current situation of nCoV.

## Data Availability

The datasets generated and/or analysed in the current study are not publicly available due to the provisions of the policy document but are available from the corresponding author on reasonable request.
