# Supplementary material for: Psychological stress and psychological support of Chinese nurses during severe public health events
Source: BMC Psychiatry. 2022 Dec 19;22:800. doi: 10.1186/s12888-022-04451-8 (PMC9761629; doi:10.1186/s12888-022-04451-8)
Supplement: Supplementary file 1 — Additional file 1. Medical staff respond to the investigation of the current situation of nCoV. [file 12888_2022_4451_MOESM1_ESM.docx]

**Medical staff respond to the investigation of the current situation of nCoV**

Dear Medical Workers,

        Thank you for your great contribution to fight against nCoV! The questionnaire is designed to conduct a mental health assessment for healthcare workers on the front lines of the fight against the nCoV, and the results of this questionnaire can provide a basis for further development of individualized psychological support and intervention plans.

        We sincerely invite you to participate in this survey, the content of this questionnaire is short, and please fill in truthfully, thank you very much. At the end of the questionnaire, you will be automatically connected to a warm mental health guide with a national psychological assistance hotline that we hope will help you.

**General information of respondents**

1. Gender [Single choice questions] *

| ○Female |
| --- |
| ○Male |

2. Age ( year): [Fill-in-the-blank questions] *

_________________________________

3. The city where you work currently: [Fill-in-the-blank questions] *

_________________________________

4. Marital status [Single choice questions] *

| ○Unmarried |
| --- |
| ○Married |
| ○Others |

5. Dwelling status [Single choice questions] *

| ○Live alone |
| --- |
| ○Not live alone |

6. Working years ( year) :_______     [Fill-in-the-blank questions] *

7. The profession of the job: [Single choice questions] *

| ○Nurse |
| --- |
| ○Doctor |
| ○Department of Imaging |
| ○Department of Laboratory |
| ○Department of Rehabilitation |
| ○Others: _________________ |

8. Highest education: [Single choice questions] *

| ○Technical secondary school |
| --- |
| ○College |
| ○Undergraduate |
| ○Master degree or above |

9. Professional title: [Single choice questions] *

| ○Not obtained |
| --- |
| ○Junior title |
| ○Intermediate title |
| ○Deputy senior title |
| ○Senior title |

**nCoV Condition of exposure**

1. Up to now, whether you have been directly involved in the diagnosis and care of someone with a fever or confirmed nCoV? [Single choice questions]*

| ○Yes |
| --- |
| ○No |
| ○Not clear |

11. How is your individual nCoV infection? [Single choice questions] *

| ○Confirmed |
| --- |
| ○Suspected |
| ○Not infected |
| ○Not clear |

12. What is the situation with nCoV infection in people you know [Single choice questions] *

| ○Someone has been diagnosed |
| --- |
| ○Someone is suspicious |
| ○Not infected |
| ○Not clear |

13. Do you have any familiar people who were diagnosed with SARS in 2003?  [Single choice questions] *

| ○Yes |
| --- |
| ○No |

14. Do you consider yourself adequately trained in COVID-19 hospital infection protection? [Single choice questions] *

| ○Yes |
| --- |
| ○No |

15. Do you think your organization is qualified to standardize personal protection in accordance with the latest "Guidelines for the Prevention and Control of Nosocomial Infections with Novel Coronavirus Infection" or relevant technical standards? [Single choice questions] *

| ○Yes |
| --- |
| ○No |

16. Do you think the current "Coronavirus Nosocomial Infection Protection Standard" will protect you from contracting the coronavirus at work? [Single choice questions] *

| ○Yes |
| --- |
| ○No |

17. Do you know the ways of psychological adjustment or counseling? [Single choice questions] *

| ○Yes |
| --- |
| ○No |

18. During this period, have you ever received psychological adjustment or counseling support? [Single choice questions] *

| ○Yes |
| --- |
| ○No |

19. How helpful do you think your current psychological adjustment or counseling support is? [Single choice questions] *

| ○Very helpful |
| --- |
| ○Helpful |
| ○General |
| ○Not very helpful |
| ○It did not help |

**Sources of stress**

20. In terms of work, do you agree that the following status quo is a source of stress[Matrix scale questions] *

|  | Strongly agree | Agree | Neutral | Disagree | Strongly disagree |
| --- | --- | --- | --- | --- | --- |
| Work tasks have increased dramatically | ○ | ○ | ○ | ○ | ○ |
| Shortage of proctective materials | ○ | ○ | ○ | ○ | ○ |
| Lack of revelant knowledge | ○ | ○ | ○ | ○ | ○ |
| The large number of patients in the ward makes cluster outbreaks more likely | ○ | ○ | ○ | ○ | ○ |
| The implementation of prevention and control measures is not good enough | ○ | ○ | ○ | ○ | ○ |
| There are unaware prevention and control loopholes | ○ | ○ | ○ | ○ | ○ |
| The hospital system is not perfect | ○ | ○ | ○ | ○ | ○ |
| Infection with nCoV due to work | ○ | ○ | ○ | ○ | ○ |

21. In terms of family, do you agree that the following status quo is a source of stress[Matrix scale questions] *

|  | Strongly agree | Agree | Neutral | Disagree | Strongly disagree |
| --- | --- | --- | --- | --- | --- |
| Unable to take care of the elderly/ children/ partner at home | ○ | ○ | ○ | ○ | ○ |
| Worry about the elderly/ Children/ partners in the family getting nCoV when they go out | ○ | ○ | ○ | ○ | ○ |
| Worry about bringing the virus/ bacteria home | ○ | ○ | ○ | ○ | ○ |
| My family does not support my work | ○ | ○ | ○ | ○ | ○ |

22. In terms of society, do you agree that the following status quo is a source of stress[Matrix scale questions] *

|  | Strongly agree | Agree | Neutral | Disagree | Strongly disagree |
| --- | --- | --- | --- | --- | --- |
| The progress of the epidemic is unknown | ○ | ○ | ○ | ○ | ○ |
| Disturbed by too many false reports. | ○ | ○ | ○ | ○ | ○ |
| Coaxing supplies | ○ | ○ | ○ | ○ | ○ |
| Being deliberately alienated by others because of my jobs | ○ | ○ | ○ | ○ | ○ |

**Generalized Anxiety Disorder 7 Item Scale（GAD-7）**

1. Over the last 2 weeks, how often have you been bothered by the following problems?[Matrix scale questions] *

|  | Not at all | | Several Days | Over Half The Days | Nearly Everyday |
| --- | --- | --- | --- | --- | --- |
| 1：Feeling nervous, anxious, or on edge | | ○ | ○ | ○ | ○ |
| 2：Not being able to stop or control worrying | | ○ | ○ | ○ | ○ |
| 3：Worrying too much about different things | | ○ | ○ | ○ | ○ |
| 4：Trouble relaxing | | ○ | ○ | ○ | ○ |
| 5：Being so restless that it’s hard to sit still | | ○ | ○ | ○ | ○ |
| 6：Becoming easily annoyed or irritable | | ○ | ○ | ○ | ○ |
| 7：Feeling afraid as if something awful might happen | | ○ | ○ | ○ | ○ |

**Percevied Stress Scale（PSS-10）**

24. The following questions ask you some of your feelings and thoughts over the past months, and for each question, please choose the one that matches you. [Matrix scale questions] *

|  | Never | Rarely | Sometimes | Often | Always |
| --- | --- | --- | --- | --- | --- |
| 1. During this period，have you been upset because of something that happened unexpectedly? | ○ | ○ | ○ | ○ | ○ |
| 1. During this period，have you felt that you were unable to control the important things in your life? | ○ | ○ | ○ | ○ | ○ |
| 1. During this period，have you felt nervous and “stressed”? | ○ | ○ | ○ | ○ | ○ |
| 1. During this period，have you felt confident about your ability to handle your personal problems? | ○ | ○ | ○ | ○ | ○ |
| 1. During this period，have you felt that things were going your way? | ○ | ○ | ○ | ○ | ○ |
| 1. During this period，have you found that you could not cope with all the things that you had to do? | ○ | ○ | ○ | ○ | ○ |
| 1. During this period，have you been able to control irritations in your life? | ○ | ○ | ○ | ○ | ○ |
| 1. During this period，you felt that you were on top of things? | ○ | ○ | ○ | ○ | ○ |
| 1. During this period，you been angered because of things that were outside your control? | ○ | ○ | ○ | ○ | ○ |
| 1. During this period，have you felt difficulties were piling up so high that you could not overcome them? | ○ | ○ | ○ | ○ | ○ |

**Social Support Rating Scale（SSRS）**

25. How many close friends, who can provide support and assistance to you do you have?[Single choice questions] *

| ○None |
| --- |
| ○One- two |
| ○Three- five |
| ○Six or more than six |

26. During the last year, you: [Single choice questions] *

| ○Are far away from your families and live alone. |
| --- |
| ○Change residence frequently and live with strangers at most of time. |
| ○Live with classmates、colleague or friends. |
| ○Live with families. |

27. During this period, you and your neighbors: [Single choice questions] *

| ○Nerve care each other and just nod when meet. |
| --- |
| ○Maybe care each other slightly while facing difficulties. |
| ○Some neighbors care you very much. |
| ○Most of neighbors care you very much. |

28. During this period, you and your colleagues: [Single choice questions] *

| ○Nerve care each other and just nod when meet. |
| --- |
| ○Maybe care each other slightly while facing difficulties. |
| ○Some care you very much. |
| ○Most of colleagues care you very much. |

29. During this period, the level of support and care that you receive from family members: [Matrix scale questions] *

|  | Never | Rarely | Generally | Fully |
| --- | --- | --- | --- | --- |
| Spouse | ○ | ○ | ○ | ○ |
| Parents | ○ | ○ | ○ | ○ |
| Children | ○ | ○ | ○ | ○ |
| Sibs | ○ | ○ | ○ | ○ |
| Other members | ○ | ○ | ○ | ○ |

30. When you met difficulties ago, the resource that you could get the economical support was/were: [Multiple-choice questions] *

| □None |
| --- |
| □Your spouse |
| □Other fammilies |
| □The friends |
| □The relatives |
| □The colleagues |
| □Your company |
| □The official or semi-official organization like the party and labor union . |
| □The non-official organization like religion and public organization. |
| □Others |

31. When you met crisis ago, the resource that you got the comfort and regard was/were: [Multiple-choice questions] *

| □None |
| --- |
| □Your spouse |
| □Other families |
| □The friends |
| □The relatives |
| □The colleagues |
| □Your company |
| □The official or semi-official organization like the party and labor union. |
| □The non-official organization like religion and public organization. |
| □Others |

32. During this period, when you are facing difficulties and feeling agonizing: [Single choice questions] *

| ○Never tell anybody. |
| --- |
| ○Just tell 1-2 people who are close to you. |
| ○Talk about it if friends ask initiatively. |
| ○Tell about it initiatively to obtain support and apprehension. |

33. During this period, the way that you ask for help when you feel annoyed: [Single choice questions] *

| ○Only lean on yourself, reject to others’ help. |
| --- |
| ○Seldom ask for others’ help. |
| ○Sometimes ask for others’ help. |
| ○Usually ask for help of the families, relatives or organizations when you meet difficulties. |

34. During this period, you join in the associations such as the party，religion organization，labor union etc. [Single choice questions] *

| ○Never |
| --- |
| ○Occassionally |
| ○Usually |
| ○Initiatively and actively |

The questionnaire is coming to an end, Subsequently, you will be automatically connected to a warm mental health guide (with a national psychological assistance hotline), and we hope it will help you. Most improtantly, We hope that you will leave a personal email address, and we will send you appropriate psychological adjustment resources by email from time to time. If needed, we are willing to continue to help us to overcome this difficult time together.

1. E-mail: [Fill-in-the-blank questions]

_________________________________
